# Siblings of Children with Autism: the Siblings Embedded Systems Framework

**DOI:** 10.1007/s40474-017-0110-5

**Published:** 2017-05-18

**Authors:** Hanna Kovshoff, Katie Cebula, Hsiao-Wei Joy Tsai, Richard P. Hastings

**Affiliations:** 1 Centre for Innovation in Mental Health, Developmental Lab, Department of Psychology, University of Southampton, Southampton, S017 1BJ UK; 2 Institute for Education, Community & Society, Moray House School of Education, The University of Edinburgh, Holyrood Road, Edinburgh, EH8 8AQ UK; 3 Centre for Educational, Development, Appraisal and Research (CEDAR), Phd, Faculty of Social Sciences, University of Warwick, Coventry, CV4 7AL UK

**Keywords:** Autism, Sibling, Well-being, Family systems, Sibling relationships, Framework

## Abstract

**Purpose of Review:**

A range of interacting factors/mechanisms at the individual, family, and wider systems levels influences siblings living in families where one sibling has autism. We introduce the Sibling Embedded Systems Framework which aims to contextualise siblings’ experience and characterise the multiple and interacting factors influencing family and, in particular, sibling outcomes.

**Recent Findings:**

Findings from studies that have reported outcomes for siblings of children with autism are equivocal, ranging from negative impact, no difference, to positive experience. This is likely due to the complex nature of understanding the sibling experience. We focus on particular elements of the framework and review recent novel literature to help guide future directions for research and practice including the influence of culture, methodological considerations, and wider participatory methods.

**Summary:**

The Siblings Embedded System Framework can be used to understand interactive factors that affect sibling adjustment and to develop clinically, educationally and empirically based work that aims to enhance and support sibling adjustment, relationships, and well-being in families of children with autism.

## Introduction

Autism is a lifelong neurodevelopmental condition characterised by pervasive impairments in social communication and repetitive or stereotyped behaviour or interests [DSM-5; 1]. The notion of an autism spectrum emphasises that each individual diagnosed with the condition experiences diverse differences across a wide range of areas of their lives. This spectrum within autism includes individual variation across measured IQ, social interaction skills, communication skills, repetitive behaviours, stereotypies, sensory needs, and adaptive skills. Typically diagnosed in early childhood, recent prevalence figures suggest that 1 in 68 individuals in the USA [2] and approximately 1 in 100 individuals in the UK [3, 4] have an autism spectrum condition. No longer a considered ‘rare’ condition, these figures highlight the need for researchers and educational/clinical practitioners to work to understand the implications of the condition for the wider family unit.

Raising a child with autism is often reported to be associated with additional strain and chronic stress in families [5]. However, it is important to note that family members of individuals with autism also report positive experiences and perceptions, and the likely causes of family members’ distress are mostly factors that may have some association with parenting a child with a disability but can be ameliorated (e.g. societal stigma, lack of social support, non-availability of professionals and supports from services, family poverty) [6]. Similarly, parents, professionals and practitioners have often assumed that siblings might be negatively affected by having a brother or sister with autism. However, existing research data are equivocal on this point, and understanding the experiences of siblings is a complex matter [6]. In the present paper, our intention is to outline a framework to help to contextualise siblings’ experiences and outcomes in families of children with autism.

To date, family systems theory, bioecological systems theory and diathesis-stress psychological frameworks have been used to understand any positive or negative influence on a child with autism and their sibling(s) as a function of a of a larger family and societal system, where intra- and inter-individual influences on the person and system are bidirectional and change over time [7–9]. In these models, no individual family member shapes the system in isolation, and the individual experiences and outcomes for any family member are dependent upon a number of systemic factors that sit both within and outside the family unit. For educators and clinicians, these models represent the practicalities and complications of ‘real-world’ work. However, at least until more recently, researchers often failed to design studies that truly took into account multiple components of a system, much less accounted for interaction between systems. For researchers, including often-uncontrollable interacting factors introduces noise and confound into the research design. Nonetheless, it is critical that researchers produce work that is truly able to provide guidance and evidence-based information for individuals and organisations that support siblings and families. Therefore, it is imperative that a more progressive research agenda is further specified and more system-focused designs and methods are adopted.

The aim of this review is to introduce a comprehensive framework for understanding the multiple and interactive factors present in families where a member has autism, with a particular focus on siblings of children with autism. Our goal is to challenge researchers and practitioners in the field (including ourselves) to move beyond a model shaped around the purported negative uni-directional impact of the child with autism on the family, towards a broader, more inclusive research agenda that incorporates a shift in thinking further towards a systems approach.

Our approach is to understand factors that have not previously been given enough focus even in more ‘enlightened’ family systems research despite a number of previous calls to explore more contextualised and systems approaches to sibling research [9, 10]. This includes research designs that do more than report on cultural, educational and socio-economic factors, but actively seek to vary samples or use population-wide data to understand the impact of these variables.

## ‘Outcomes’ for Siblings of Children with Autism—What Do We Know?

We do not intend to provide a comprehensive overview of research findings about the experiences and outcomes for siblings of children with autism. Other excellent reviews of the extant literature on siblings of children with autism exist [11]. Instead, we intend to illustrate some key points and, with a degree of ‘artistic license’, report the existing research literature necessarily briefly and with limited regard to detail and nuance.

Although parents often report concerns about the siblings of children with autism, perhaps due to increased care responsibilities or reduced parental attention, data on siblings’ well-being suggests that a negative impact hypothesis is ‘not proven’. We would argue strongly that a negative impact hypothesis is not proven in at least four key ways. First, some studies suggest that siblings of children with autism have more behavioural and emotional problems when compared with other siblings or other children generally [12]. Some find no such differences in levels of problems [13], and indeed, some studies suggest that siblings of children with autism are better adjusted than comparison groups of children [14]. Second, even when there are suggestions of negative impact on siblings, the numbers of siblings with concerning levels of behavioural and emotional problems are often very small [15]. Thus, negative impact on siblings’ own adjustment is not universal. Third, estimates of any negative impact vary quite considerably with who is asked. Mothers may report that siblings have higher levels of problems than do fathers [16], who both report that siblings have higher levels of problems than do siblings themselves [17]. Fourth, richer reports of the sibling experience derived from qualitative studies make clear that siblings have positive experiences with their brothers and sisters with autism [18].

Thus, existing research findings suggest that some siblings of children with autism do experience more problems of psychological adjustment than other children, but negative impact is by no means inevitable or universal. It is important to also recognise positive perspectives and putative positive impact [6]. In addition, only a relatively small number of research studies have examined what it is about living with a brother or sister with autism that could have a negative impact on siblings. A key variable is the level of behaviour problems of the child with autism; longitudinal research designs [19] have suggested that later sibling problems are associated with earlier, higher levels of behaviour problems of the brother/sister with autism. Siblings of children with autism who also have behaviour problems may be a high-risk group for problems themselves. Somewhat less studied, but potentially important, is that the broader autism phenotype (BAP), or the presence of elevated autistic traits, may be associated with sibling adjustment problems in addition to, or instead of, the direct impact of the child with autism [8, 20–22].

This brief section has focused only on the well-being/psychological adjustment of siblings of children with autism. This is mainly because the putative negative impact of children with autism on their siblings directly (e.g. through behaviour problems) or indirectly (e.g. through parents’ attention being needed for the child with autism) has been a dominant question in autism sibling research to date. Even considering this one core question about siblings’ experiences reveals the complexity of the issue—there are some specific risk factors, and there are significant variations by demographic and family factors (e.g. family size, access to support), with findings affected by the choice of methodology and research design. To understand this variation in relation to sibling adjustment outcomes, but also other dimensions of siblings’ lives, requires careful thought built on some strong theoretical foundations.

## A Framework to Continue Forward Momentum in Sibling Research

**Fig. 1:**
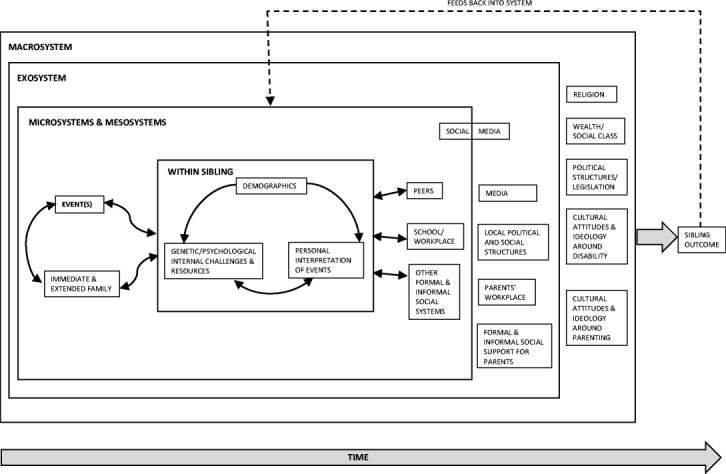
Sibling Embedded Systems Framework

Here, we introduce the ‘Sibling Embedded Systems Framework’ (Fig. 1). This framework builds upon existing theory and empirical research in the field. It incorporates elements of family systems theory [23], the Double ABCX model [24], the diathesis-stress model [25] and the bioecological systems model [26, 27]. Individually, several of these theoretical models have been well used in autism family research to date; conceptualisations of the family as a holistic, dynamic and integrated system have been invaluable in guiding research to better understand the experiences and outcomes of parents [28–30] and of siblings [7]. More recently, diathesis-stress model exploration has begun into the interplay between genetic and environmental risk factors for the sibling [8]. The bioecological model, which posits the need to understand the developing child in the context of the environmental settings which they experience directly and indirectly, has been less commonly employed, despite the advantages that this offers in understanding the broader contextual factors affecting siblings’ lives [9, 10]. Whilst such recent research does suggest that the field has moved away from its previous largely ‘atheoretical’ stance [9], there has been little attention given explicitly to synthesising theoretical frameworks from a wider standpoint that combines individual and broader system perspectives. Existing theoretically informed models are also not explicitly designed with a focus on sibling relationships [for an exception and comprehensive overview, see 10]. Many of the pathways shown in our framework have good empirical support, as discussed below, though others have been less thoroughly explored.

The Sibling Embedded Systems Framework is not specific to disability research, but lends itself to the particular context of understanding siblings of children with autism. Adopting the bioecological systems approach [26, 27], it incorporates a series of nested levels, from micro-systems directly experienced by the sibling, the meso-system interplay between these factors, through the exosystem in which the sibling does not directly participate, but is nonetheless affected by, to broader societal and cultural macro-system factors. Within this framework, the child is not viewed as passive, but as actively shaping their environment. Additional genetic, child and family factors, drawn from the other key theories [23–25], are included to produce a more detailed and complete framework for understanding sibling experiences.

Figure 1 illustrates some of the key factors at each level. Within the micro- and meso-system levels, one of the key factors is the ‘event’. This event may be the child with autism, but unlike much family systems work, the framework does not position the child with autism as a ‘stressor’ or ‘demand’ on the family. The use of this more neutral term of ‘event’ both supports the framework’s application in research into the positive impact of the child with autism on the family [31] and also emphasises variability in impact over time. Further, the ‘event’ may not be the child with autism, but instead the presence of a neurotypical child in the family, which sits well with recent research that has focused on the sibling relationship from the perspective of the child with autism [32]. Equally, the ‘event’ might be a specific aspect of the child with autism’s developmental profile, such as behaviour problems (see earlier) or communication skills. The term ‘event’ also allows for research into more detailed moments of family life, such as the receipt of a diagnosis for the child with autism or a school transition. Such events have naturally been examined, to date, predominately from the perspective of the child with autism and their parents, but given reports of the negative impact of these life events [33], knock-on effects on siblings should also be explored. It is possible too that ‘the event’ in fact covers multiple events, such as the presence of a child with autism alongside life events such as a bereavement. Whilst this is similar to the notion of ‘pile-up demands’ in family system research [5], in the present framework again, such situations are not labelled, a priori, as negative.

The ‘event’ is then theorised to impact on the sibling, but this impact will differ according to ‘within-sibling’ factors: demographic variables, internal challenges and resources and the sibling’s interpretation of events. Demographic factors such as gender, gender match between siblings and age relative to the child with autism have all been shown to play a role in sibling outcomes [34], and whilst findings have not always been consistent, there is support for the suggestion that the overall number of demographic ‘risk’ factors is important in predicting sibling outcomes [22].

‘Internal challenges’ refer to any difficult within-sibling factor. This might include, for example, a language impairment or avoidant coping strategies [35]. ‘Internal resources’ refer to any uplifting or helpful within-sibling factor. This might include, for example positive self-esteem, resilience, or adaptive coping skills [36]. In some cases, it may be difficult to know in advance whether a factor is a challenge or a resource. For example, the BAP might be assumed to be a challenge, given that it has been associated with poorer outcomes for siblings of children with autism (see earlier). However, it is also possible that in certain contexts, the BAP will operate as a resource; for example, if there is increased affinity between children with autism and high-BAP siblings, because of similar interests or interaction styles, this will potentially result in more harmonious sibling relationships. Other internal factors, such as temperament and personality, are similarly not well understood [though see 37].

The final suggested within-sibling factor is the sibling’s own interpretation of the event, with more positive interpretations predicted to be associated with more positive outcomes, as has been found with parents of children with autism [5, 30]. Interactions between these three ‘within-sibling’ factors—demographic variables, challenges/resources and event interpretation—are also predicted, though the pattern of such interactions may change as the sibling ages.

Within the micro- and meso-level of the framework, sibling outcomes are affected not only by ‘the event’ but also by factors within the immediate and extended family. Within the immediate family, such factors have been found to include demographic characteristics [38], parental mental health [20, 39], parental BAP [21, 39] and parent relationship quality [37]. The influence of family interactions on sibling outcomes has also been explored to some extent, including work into parenting style [40], differential parenting [41] and sibling relationship quality [42]. Extended family may also be a key influence, either directly on the sibling, or indirectly via support for, or conflict with the parents. However, whilst there has been a small amount of research with grandparents of children with autism [43], to date, this has not involved siblings. Similarly, the influence of more complex, but common family relationships, such as several siblings with autism, stepsiblings and non-resident parents, is little understood.

For other factors within the micro- and meso-systems, such as peers, the sibling’s school/workplace and support systems, existing research evidence is somewhat limited in relation to siblings of children with autism. Although there is good evidence that social support is associated with more positive outcomes for siblings [44–46], other types of peer influence and changes across the sibling’s lifespan are not fully understood. There has been recent work in this area, for example Stampoltzis et al. [47] explored the responses of the sibling’s peers to the child with autism and found that more positive responses were associated with higher levels of self-esteem in the sibling. Nowell et al. [48] explored peer teasing/bullying of siblings, but were not able to establish whether this teasing related specifically to being a sibling of a child with autism, nor how it related to sibling outcomes. The influence of school factors, such as both siblings attending the same school, inclusive school attitudes, or a sense of school connectedness, has similarly not been well explored, though such school ethos is increasingly recognised as important for the child with autism [49]. Similarly, whilst employment status has been explored as an outcome measure for adult siblings [e.g. 50], the wider role of the workplace for adult siblings (e.g. in offering a flexible work environment to facilitate caregiving of the autistic family member) is not well explored. Additionally, little is known about the role of social media in which the sibling directly participates, for example as a source of social support or indeed of additional challenge.

The relationships shown using arrows in Fig. 1 seem to suggest bidirectional, but linear, relationships between the various elements in the micro- and meso-systems. To date, bidirectional pathways have seldom been explored [though see 19, 51]. Pathways within the framework will also be characterised by more complex patterns of mediation and moderation. A recent example here involves the diathesis-stress model, showing how genetic liability (e.g. sibling BAP) may interact with environmental stressors (e.g. behaviour problems of the child with autism) to impact outcomes for the sibling [8, 20, 21, 52]. Indeed, this work is part of a wider literature recognising the value of mediation and moderation analysis to better understand the influences on sibling outcomes [14, 45, 53].

We do not, at present, have a complete picture of the interplay between factors at the micro-system level and the extent to which these influence outcomes for the sibling across their lifespan. The mesosytem, e.g. interactions between the sibling’s home and school environments, is also neglected [10]. Nonetheless, it is clear from the research drawn upon here that much progress has been made in terms of understanding the child’s immediate environment. In contrast, within the exo- and macro-system levels, the research evidence is sparse. Of the factors at the exo-system level, social support received by the parents is perhaps the best understood in terms of its influence on sibling adjustment. For example, Cebula [44] found that social support received by parents was associated with better sibling adjustment in families using early autism interventions. Similarly, Hastings [14] found that family social support moderated the impact of symptom severity in the child with autism on sibling adjustment.

Culture is one example of a macro-system factor found to be associated with sibling outcome [e.g. 21]. ‘Culture’ refers to the ideas, customs and behaviour of a particular group of people or a society [54], the values that people believe in or traditions that they follow; therefore, culture can permeate the ways people understand and behave in any given situation. In this sense, people’s lives are influenced by the collective attitudes and behavioural characteristics of their society on a number of different levels.

In the case of families, culture can influence micro-system factors such as parenting styles and children’s behaviour towards their parents [55, 56]. For families with a child with disabilities, it can also affect the ways in which they seek help, relate to professionals and approach interventions, as well as the coping patterns they adopt [57]. Culturally informed beliefs about the cause of disability may also have consequences for action and treatment approaches [58]. ‘Culture’ can be difficult to measure, though race, ethnicity or nationality is often used as a proxy measure [58]. Even within a single country, there can be great variation, with race and ethnicity-related disparities in access to healthcare for children with autism [59].

Whilst there are, at present, relatively few cross-cultural studies involving families of children with autism, not unexpectedly, there is some evidence of cross-cultural similarities in family experience. For example, positive associations between social support and sibling adjustment have been found in both Taiwan and UK families [7]. There is evidence too of cross-cultural differences, for example in the coping strategies used by Taiwanese and American mothers of children with autism [60]. Similarly, Tsai, Cebula and Fletcher-Watson [21], using a diathesis-stress model, reported differences between Taiwanese and UK families in terms of the specific sibling outcomes found to be significantly associated with interactions between sibling BAP and environmental factors. In the UK families, the interaction of sibling BAP with environmental stress (severity of symptoms in the child with autism) correlated with the siblings’ behaviour problems (total difficulties and peer problems), but in the Taiwanese families, it was siblings’ prosocial behaviour that was affected. Despite such examples, little progress has been made in research on the cultural factors that shape the experiences of siblings of children with autism. Most studies focus mainly on Euro-American cultural contexts and are limited only to cases within one country [53].

Other broad exo- and macro-system factors such as local political structures, media, religion and legislation are similarly seldom the focus of research. The impact of growing up in an economically disadvantaged community is a particularly important aspect that remains neglected in research. Little is known too of the pathways between macro-system factors and sibling outcomes. Research on culture [e.g. 21] has explored between-country differences in sibling outcome, which are not posited to result from macro-system cultural differences between the two countries. However, the specific pathways from macro-system ‘culture’ to sibling outcome are rather poorly understood at present, and work is hampered by methodological issues such as a lack of culturally sensitive measures of sibling outcomes. Detailed knowledge of the pathways from other macro-system factors (e.g. legislation, religion) to sibling outcomes is also lacking. One reason for this may be that when influential factors are several steps removed from the child (e.g. the sibling), it becomes increasingly difficult to ascertain whether and how they are associated with child outcomes [61]. Identification of more proximal potential mediators can be helpful so that hypothesised ‘causal chains’ from macro influence to child outcome can be explored [61].

Two final elements of the framework worth highlighting are the sibling outcome and the notion of time. Sibling outcomes include psychosocial adjustment, sibling relationship quality, academic achievement, or life satisfaction/quality of life, with some factors, such as sibling relationship quality and self-esteem, operating as both predictor and outcome variables. Within the framework, sibling outcomes are hypothesised to feedback into the system. For example, siblings with more positive emotional/behavioural outcomes are likely to interact differently with the child with autism than would siblings experiencing difficulties. Indeed, sibling behaviour problems have been shown to predict the behaviour problems of the child with autism over time [51]. The notion of time is also important, with few of the factors within the framework remaining constant across the sibling’s lifespan, and early experiences shaping later outcomes, emphasising the importance of longitudinal research designs. Due regard is needed, though, for the temporal validity of findings, given that siblings today are developing in a very different cultural environment from siblings in previous generations [62]. Whilst some factors, such as parenting styles and coping strategies, may have a degree of longevity in terms of their influence on sibling outcomes, other aspects of siblings’ lives, such as social media, may change more rapidly from one generation to the next, suggesting the need for caution when using older research findings to inform current approaches to understanding and supporting siblings.

In sum, the development of this theoretical framework is our attempt to answer the call for greater use of theory to guide this research field [9, 62]. Clearly, it would be impossible to include all elements of the Sibling Embedded Systems Framework in any single research study. Instead, it is necessary to build up a fuller picture using individual studies, systematic reviews and intervention designs and working directly with siblings and their families to identify priority research areas. As is clear from the discussion above, a greater focus on exo- and macro-system elements in particular is required.

## Methodological Developments

Calls for methodological developments to improve the sibling research field have been made repeatedly [9, 53, 62]. There have been encouraging developments in autism sibling research, including research with more diverse groups of participants [63], larger samples [22, 39, 64], adult siblings [50], multiple informants—including the sibling and the child with autism themselves [17, 32]—and longitudinal designs [19, 51, 65]. However, high-quality research from a methodological and design perspective is not common, perhaps hampered by the time and costs involved in such studies. Given the typical research participant profile to date, there continues to be a need for further research with neglected groups, including economically disadvantaged families and non-white, non-Western participants. Research with siblings and parents who are not neurotypical, including those who themselves have autism, is also required [see, e.g. 66].

Much child research is increasingly moving to the use of ‘big data’, with analysis of cohort data such as the Millennium Cohort Study [67] and the Growing up in Scotland study [68] helping to understand the role of broader macro-system factors on child outcomes. Where datasets identify siblings of children with autism, they offer great advantage in overcoming the methodological drawbacks associated with non-representative samples. However, such datasets may include data on only a limited number of families of children with autism [only a few over 100 in the Millennium Cohort study for example; 69] and few sibling variables [22, 39]. Hence, larger-scale family studies including detailed measurement of variables across all system levels identified in Fig. 1 are also required to develop our understanding of the role of the macro-system ‘big picture’ in sibling outcomes. More difficult to explore, but also of interest, is the extent to which siblings, rather than being passively influenced by these macro-system factors, may actively shape their own wider environment, for example by contributing, even in a small way, to cultural attitudes towards disability.

Following moves in autism research more broadly [70], a wider participatory framework should be used so that the sibling research agenda reflects the views and priorities of the broader population we are aiming to understand and support. Within this, it is imperative that the voice of the child with autism is both sought and heard, regardless of ‘level of functioning’, that siblings who are not ‘typically developing’ are included in this discussion and that individuals from families from a wider range of cultural and socio-economic backgrounds are provided the opportunity to influence and shape research and practice agendas. Such consultation should inform not only research into family experiences, but also the design and evaluation of sibling support interventions.

## Conclusions

A key aim, in the development of the Sibling Embedded Systems Framework outlined here, is to stimulate theoretically driven sibling research which will ultimately enhance family and sibling interventions and support. However, as McHale et al. [71] noted, those supporting siblings of children with autism cannot wait for all aspects of theoretical models to be empirically explored before interventions to enhance sibling adjustment and relationships are developed. It is clear from the discussion above that there is now a large body of sibling research, and this has enabled the beginnings of theory-informed interventions [72], though interventions are not always evidence-based [73]. Indeed, evaluations of interventions may produce data that help to refine sibling theoretical models and frameworks.

We believe that the Sibling Embedded Systems Framework has the potential to guide research, ultimately leading to a better understanding of factors that affect siblings, and an understanding of how different siblings are best supported at different times and in different contexts. Only with a seismic shift in our thinking, in our practice and in our research design can we begin to truly answer questions about outcomes for families, where one member has a lifelong neurodevelopmental condition.

## References

[1] American Psychiatric Association (2013). Diagnostic and statistical manual of mental disorders: DSM-5.

[2] CDC (2013). Prevalence of autism spectrum disorders among children aged 8 years: autism and developmental disabilities monitoring network, 11 sites, United States, 2010. MMWR Surveill Summ.

[3] Baird G, Simonoff E, Pickles A, Chandler S, Loucas T, Meldrum D, Charman T (2006). Prevalence of disorders of the autism spectrum in a population cohort of children in the South Thames: the special needs and autism project (SNAP). Lancet.

[4] Brugha T, Mcmanus S, Bankart J, Scott F, Purdon S, Smith J, Meltzer H (2011). Epidemiology of autism spectrum disorders in adults in the Community in England. Arch Gen Psychiatry.

[5] McStay RL, Trembath D, Dissanayake C (2014). Stress and family quality of life in parents of children with autism spectrum disorder: parent gender and the double ABCX model. J Autism Dev Disord.

[6] Hastings RP (2016). Do children with intellectual and developmental disabilities have a negative impact on other family members? The case for rejecting a negative narrative. International Review of Research in Developmental Disabilities.

[7] Tsai HWJ, Cebula K, Fletcher-Watson S (2016). Influences on the psychosocial adjustment of siblings of children with autism spectrum disorder in Taiwan and the United Kingdom. Research in Autism Spectrum Disorders..

[8] Orsmond GI, Seltzer MM (2009). Adolescent siblings of individuals with an autism spectrum disorder: testing a diathesis-stress model of sibling well-being. J Autism Dev Disord.

[9] Stoneman Z (2005). Siblings of children with disabilities: research themes. J Inf Secur.

[10] Saxena M, Adamsons K (2013). Siblings of individuals with disabilities: reframing the literature through a bioecological lens. Journal of Family Theory & Review.

[11] Meadan H, Stoner JB, Angell ME (2010). Review of literature related to the social, emotional, and behavioral adjustment of siblings of individuals with autism spectrum disorder. J Dev Phys Disabil.

[12] Fisman S, Wolf L, Ellison D, Gillis B, Freeman T, Szatmari P (1996). Risk and protective factors affecting the adjustment of siblings of children with chronic disabilities. J Am Acad Child Adolesc Psychiatry.

[13] Pilowsky T, Yirmiya N, Doppelt O, Gross-Tsur V, Shalev RS (2004). Social and emotional adjustment of siblings of children with autism. J Child Psychol Psychiatry.

[14] Hastings RP (2003). Behavioral adjustment of siblings of children with autism engaged in applied behavior analysis early intervention programs: the moderating role of social support. J Autism Dev Disord.

[15] Petalas MA, Hastings RP, Nash S, Lloyd T, Dowey A (2009). Emotional and behavioural adjustment in siblings of children with intellectual disability with and without autism. Autism: The International Journal of Research and Practice..

[16] Griffith GM, Hastings RP, Petalas MA (2014). Brief report: fathers’ and mothers’ ratings of behavioral and emotional problems in siblings of children with autism spectrum disorder. J Autism Dev Disord.

[17] Hastings RP, Petalas MA (2014). Self-reported behaviour problems and sibling relationship quality by siblings of children with autism spectrum disorder. Child Care Health Dev.

[18] Petalas MA, Hastings RP, Nash S, Reilly D, Dowey A (2012). The perceptions and experiences of adolescent siblings who have a brother with autism spectrum disorder. J Intellect Dev Disabil.

[19] Hastings RP (2007). Longitudinal relationships between sibling behavioral adjustment and behavior problems of children with developmental disabilities. J Autism Dev Disord.

[20] Petalas MA, Hastings RP, Nash S, Hall LM, Joannidi H, Dowey A (2012). Psychological adjustment and sibling relationships in siblings of children with autism spectrum disorders: environmental stressors and the broad autism phenotype. Research in Autism Spectrum Disorders.

[21] Tsai, H. W. J. Cebula, K., & Fletcher-Watson, S. The role of the broader autism phenotype and environmental stressors in the adjustment of siblings of children with autism spectrum disorders in Taiwan and the United Kingdom. Journal of Autism and Developmental Disorders. 2017. https://sp.ukdataservice.ac.uk/doc/7432/mrdoc/pdf/7432_gus_bc2_s1_user_guide.pdf. 10.1007/s10803-017-3134-0 PMC5509828 28502037

[22] Walton K (2016). Risk factors for behavioral and emotional difficulties in siblings of children with autism spectrum disorder. American Journal on Intellectual and Developmental Disabilities.

[23] Minuchin S (1974). Families and family therapy.

[24] McCubbin HI, Patterson JM (1983). The family stress process—the double ABCX model of adjustment and adaptation. Marriage Fam Rev.

[25] Bauminger N, Yirmiya N, Burack JA, Charman T, Yirmiya N, Zelazo PR (2001). The functioning and well-being of siblings of children with autism: behavioral-genetic and familial contributions. The development of autism: perspectives from theory and research.

[26] Bronfenbrenner U (1979). The ecology of human development: experiments by nature and design.

[27] Bronfenbrenner U (2005). Making human beings human: bioecological perspectives on human development.

[28] Bristol M (1987). Mothers of children with autism or communication disorders: successful adaptation and the double ABCX model. J Autism Dev Disord.

[29] Cridland EK, Jones SC, Magee CA, Caputi P (2014). Family-focused autism spectrum disorder research: a review of the utility of family systems approaches. Autism: The International Journal of Research and Practice..

[30] Pozo P, Sarriá E, Brioso A (2014). Family quality of life and psychological well-being in parents of children with autism spectrum disorders: a double ABCX model. J Intellect Disabil Res.

[31] Taunt HM, Hastings RP (2002). Positive impact of children with developmental disabilities on their families: a preliminary study. Educ Train Ment Retard Dev Disabil.

[32] Petalas MA, Hastings RP, Nash S, Duff S (2015). Typicality and subtle difference in sibling relationships: experiences of adolescents with autism. J Child Fam Stud.

[33] Makin C, Hill V, Pellicano E (2017). The primary to secondary school transition for children on the autism spectrum: a multi informant mixed methods study. Autism and Developmental Language Impairments.

[34] Macks RJ, Reeve RE (2007). The adjustment of non-disabled siblings of children with autism. J Autism Dev Disord.

[35] Roeyers H, Mycke K (1995). Siblings of a child with autism, with mental retardation and with a normal development. Child Care Health Dev.

[36] Orsmond GI, Kuo HY, Seltzer MM (2009). Siblings of individuals with an autism spectrum disorder: sibling relationships and wellbeing in adolescence and adulthood. Autism: The International Journal of Research and Practice.

[37] Rivers JW, Stoneman Z (2008). Child temperaments, differential parenting, and the sibling relationships of children with autism spectrum disorder. J Autism Dev Disord.

[38] Kaminsky L, Dewey D (2002). Psychosocial adjustment in siblings of children with autism. J Child Psychol Psychiatry.

[39] Shivers CM, Deisenroth LK, Taylor JL (2013). Patterns and predictors of anxiety among siblings of children with autism spectrum disorders. J Autism Dev Disord.

[40] Gau SS, Chou MC, Lee JC, Wong CC, Chou WJ, Chen MF, Wu YY (2010). Behavioral problems and parenting style among Taiwanese children with autism and their siblings. Psychiatry Clin Neurosci.

[41] Rivers J, Stoneman W (2003). Sibling relationships when a child has autism: marital stress and support coping. J Autism Dev Disord.

[42] Tomeny TS, Ellis BM, Rankin JA, Barry TD (2017). Sibling relationship quality and psychosocial outcomes among adult siblings of individuals with autism spectrum disorder and individuals with intellectual disability without autism. Res Dev Disabil.

[43] D’Astous V, Wright SD, Wright CA, Diener ML (2013). Grandparents of grandchildren with autism spectrum disorders: influences on engagement. Journal of Intergenerational Relationships.

[44] Cebula KR (2012). Applied behavior analysis programs for autism: sibling psychosocial adjustment during and following intervention use. J Autism Dev Disord.

[45] Tomeny T, Barry T, Fair E. Parentification of adult siblings of individuals with autism spectrum disorder: distress, sibling relationship attitudes, and the role of social support. J Intellect Develop Disabil. 2016:1–12.

[46] Wolf L, Fisman S, Ellison D, Freeman T (1998). Effect of sibling perception of differential parental treatment in sibling dyads with one disabled child. Journal of the American Academy of Child & Adolescent Psychiatry.

[47] Stampoltzis A, Defingou D, Antonopoulou K, Kouvava S, Polychronopoulou S. Psycho-social characteristics of children and adolescents with siblings on the autistic spectrum. European Journal of Special Needs Education. 2014;29(4):474–90.

[48] Nowell KP, Brewton CM, Goin-Kochel RP (2014). A multi-rater study on being teased among children/adolescents with autism spectrum disorder (ASD) and their typically developing siblings: associations with ASD symptoms. Focus on Autism and Other Developmental Disabilities.

[49] Hebron J, Little C (2017). The transition from primary to secondary school for students with autism spectrum disorders. Supporting social inclusion for students with autism spectrum disorders.

[50] Howlin P, Moss P, Savage S, Bolton P, Rutter M (2015). Outcomes in adult life among siblings of individuals with autism. J Autism Dev Disord.

[51] Hastings RP, Petalas MA, Jones L, Totsika V (2014). Systems analysis of associations over time between maternal and sibling well-being and behavioral and emotional problems of children with autism. Research in Autism Spectrum Disorders..

[52] Walton KM, Ingersoll BR (2015). Psychosocial adjustment and sibling relationships in siblings of children with autism spectrum disorder: risk and protective factors. J Autism Dev Disord.

[53] Hodapp RM, Glidden LM, Kaiser AP (2005). Siblings of persons with disabilities: toward a research agenda. Ment Retard.

[54] Berry JW, Poortinga YH, Breugelmans SM, Chasiotis A, Sam DL (2011). Cross-cultural psychology research and applications.

[55] Chao R (1994). Beyond parental control and authoritarian parenting style: understanding Chinese parenting through the cultural notion of training. Child Dev.

[56] Ho DY, Bond MH (1996). Filial piety and its psychological consequences. The andbook of Chinese psychology.

[57] Ravindran N, Myers BJ (2011). Cultural influences on perceptions of health, illness, and disability: a review and focus on autism. J Child Fam Stud.

[58] Mandell DS, Novak M (2005). The role of culture in families’ treatment decisions for children with autism spectrum disorders. Ment Retard Dev Disabil Res Rev.

[59] Liptak GS, Benzoni LB, Mruzek DW, Nolan KW, Thingvoll MA, Wade CM, Fryer GE (2008). Disparities in diagnosis and access to health services for children with autism: data from the National Survey of Children’s Health. Journal of Developmental and Behavioural Pediatrics.

[60] Lin LY, Orsmond GI, Coster WJ, Cohn ES (2011). Families of adolescents and adults with autism spectrum disorders in Taiwan: the role of social support and coping in family adaptation and maternal well-being. Research in Autism Spectrum Disorders..

[61] Romich JL (2006). Randomized social policy experiments and research on child development. J Appl Dev Psychol.

[62] Orsmond GI, Seltzer MM (2007). Siblings of individuals with autism spectrum disorders across the life course. Ment Retard Dev Disabil Res Rev.

[63] Manning M, Wainwright M, Bennett L (2011). The double ABCX model of adaptation in racially diverse families with a school-age child with autism. J Autism Dev Disord.

[64] Dempsey AG, Llorens A, Brewton C, Mulchandani S, Goin-Kochel RP (2012). Emotional and behavioral adjustment in typically developing siblings of children with autism spectrum disorders. J Autism Dev Disord.

[65] Fisman S, Wolf L, Ellison D, Gillis B, Freeman T (2000). A longitudinal study of siblings of children with chronic disabilities. Can J Psychiatr.

[66] Pohl, A. L., Crockford, S.K., Allison, C., & Baron-Cohen, S. Positive and negative experiences of mothers with autism. Paper presented at the International Meeting for Autism Research, Baltimore, M.D. 2016.

[67] Hansen K (2010). Millennium cohort study first, second, third and fourth surveys: a guide to the datasets.

[68] Bradshaw P, Corbett J (2013). Growing up in Scotland: birth cohort 2, sweep 1, user guide.

[69] Totsika V, Hastings RP, Emerson E, Berridge DM, Lancaster GA (2011). Behavior problems at 5 years of age and maternal mental health in autism and intellectual disability. J Abnorm Child Psychol.

[70] Pellicano E, Dinsmore A, Charman T (2014). What should autism research focus upon? Community views and priorities from the United Kingdom. Autism: The International Journal of Research and Practice..

[71] McHale SM, Updegraff KA, Feinberg ME (2016). Siblings of youth with autism spectrum disorders: theoretical perspectives on sibling relationships and individual adjustment. J Autism Dev Disord.

[72] Tsao LL, Davenport R, Schmiege C (2012). Supporting siblings of children with autism spectrum disorders. Early Childhood Educ J.

[73] Tudor M, Lerner E (2015). Intervention and support for siblings of youth with developmental disabilities: a systematic review. Clin Child Fam Psychol Rev.

